# Accommodate or Reject: The Role of Local Communities in the Retention of Health Workers in Rural Tanzania

**DOI:** 10.34172/ijhpm.2021.77

**Published:** 2021-07-26

**Authors:** Nathanael Sirili, Daudi Simba, Joseph M Zulu, Gasto Frumence, Moses Tetui

**Affiliations:** ^1^Department of Development Studies, School of Public Health and Social Sciences, Muhimbili University of Health and Allied Sciences, Dar es Salaam, Tanzania.; ^2^Department of Community, School of Public Health and Social Sciences, Muhimbili University of Health and Allied Sciences, Dar es Salaam, Tanzania.; ^3^School of Public Health, University of Zambia (UNZA), Lusaka, Zambia.; ^4^School of Pharmacy, University of Waterloo, Waterloo, ON, Canada.; ^5^Department of Epidemiology and Global Health, Umeå University, Umeå, Sweden.; ^6^Department of Health Policy, Planning and Management, Makerere University School of Public Health, Kampala, Uganda.

**Keywords:** Health Workforce Retention, Primary Health Care, Community, Rural, Tanzania

## Abstract

**Background:** While over 70% of the population in Tanzania reside in rural areas, only 25% of physicians and 55% of nurses serve these areas. Tanzania operates a decentralised health system which aims to bring health services closer to its people through collaborative citizen efforts. While community engagement was intended as a mechanism to support the retention of the health workforce in rural areas, the reality on the ground does not always match this ideal. This study explored the role local communities in the retention of health workers in rural Tanzania.

**Methods:** An exploratory qualitative study was completed in two rural districts from the Kilimanjaro and Lindi regions in Tanzania between August 2015 and September 2016. Nineteen key informant interviews (KIIs) were conducted with district health managers, local government leaders, and health facility in-charges. In addition, three focus group discussions (FGDs) were conducted with 19 members of the governing committees of three health facilities from the two districts. Data were analysed using the thematic analysis technique.

**Results:** Accommodation or rejection were the two major ways in which local communities influenced the quest for retaining health workers. Communities accommodated incoming health workers by providing them a good reception, assuming responsibility for resolving challenges facing health facilities and health workers, linking health workers to local communities and promoting practices that placed a high value on health workers. On the flip side, communities could also reject health workers by openly expressing lack of trust and labelling them as ‘foreigners,’ by practicing cultural rituals that health workers feared and discrimination based on cultural differences.

**Conclusion:** Fostering good relationships between local communities and health workers may be as important as incentives and other health system strategies for the retention of health workers in rural areas. The role communities play in rural health worker retention is not sufficiently recognized and is worthy of further research.

## Background

**Key Messages**

**Implications for policy makers**

- In designing health worker retention strategies, policy-makers should factor in the relationship between local communities and health workers.
- Interventions targeting community actions that promote attraction and retention of health workers in rural communities should be developed.
- Priority should be given to large scale research on the context specific roles that communities can play in retention of health workers.

**Implications for the public**

Attracting and retaining health workers to rural areas of Tanzania is a major priority. This research has documented the powerful roles that communities can play in supporting or discouraging health workers to stay in rural areas. Strategies are needed that better harness the positive potential of communities in rural health worker retention. Conversely, this study has unveiled the consequences of community actions on the retention of health workers coming from different cultural backgrounds. Last but not least, this study has opened a ‘pandora’s box’ of cultural rituals which differ from one community to another and how these can be viewed as sensitive to people coming from a different community.

Retention of the skilled health workforce in rural and remote areas has remained a hurdle to many health systems globally.^1,2^ By 2015, only 38% of nurses and 24% of physicians served the rural population.^3^ The available literature points to work and living environments, low remuneration, lack of economic opportunities and limited career prospects as the major factors underlying the poor retention of the health workforce in rural areas.^4-6^ Despite the understanding that the retention of health workers in rural areas is a multifaceted problem, strategies tend to be fragmented and selective in their approaches, such as the provision of financial incentives or specific career opportunities.^7^ These ‘supply side’ approaches also do not consider or engage the actions of local communities and their influence on the retention of health workers.

Tanzania, a middle-income country in sub-Saharan Africa is not an exception in the quest for the retention of health workers in rural areas.^8^ While over 70% of the population resides in rural areas,^10^ only 55% of nurses and 25% of physicians work in rural areas.^11^ In the context of this study, health workers refer to the formally trained, recruited and deployed health workers of the government of Tanzania who are officially recognized in the public service scheme and staffing norms of the Ministry of Health and Social Welfare.^9^ Like many other countries, Tanzania has had a long history of attempts to address health workforce challenges. The health sector reforms of the 1990s stand out as one of the major efforts aimed at improving health workforce retention in Tanzania.

The 1990s health sector reforms firstly reversed a 1977 ban on the private provision, allowing for private practices, opening of private health facilities, opening private health training institutions, and re-engaging this sector through public private partnerships.^13^ Secondly, the reforms re-introduced decentralized health system administration in the form of devolution.^12^ Tanzania adopted a decentralised health system with three levels; the primary, secondary and tertiary level.^14^ The primary level health system was made the focal point for healthcare services planning, provision and programme implementation^12^ and the districts were given the mandate to recruit, deploy and retain health workers.^15^ To fulfil the new roles of devolved administration and the goal of ‘bringing services closer to the people,’ community engagement at all levels of decision making and implementation was made mandatory. Community health workers (CHWs) were deployed to improve access to healthcare services. Although not a formalised cadre, CHWs are considered a core component of community-based health systems^16^ linking local communities to health facilities. In addition to CHWs, Health Facility Governing Committees (HFGCs) were formed to ensure that the community is adequately and systematically engaged. The HFGCs are comprised of five elected community members and three appointed members (the health facility in-charge, a member from the village government committee, and a member of the Ward Development Committee). The main roles of the HFGCs include (*i*) developing the plans and budget of the facility and (*ii*) liaising with the Health Facilities Management Teams and other actors to ensure the delivery of quality health services.^17^

In addition to the administrative reforms described above, the government of Tanzania has tried several specific strategies combining both financial and non-financial incentives to retain the skilled health workforce in rural areas. However, these measures have produced little or no observable improvements.^18^ Financial incentives have often failed to materialise due to budget limitations while most of the non-financial incentives have also required some form of funding. Non-financial incentives to the health workers may appear to be cheap, but can have huge financial costs to the government and may thus be difficult to implement.^19^ Non-financial incentives in Tanzania have included: opportunities for education career development, proactive staff recruitment, compulsory community service, bonding schemes, contracting arrangements and provision of accommodation.^12,20,21^ Most of these strategies have been proposed through the central government with little or no involvement of the decentralised health system and without being piloted. This has created challenges such as poor understanding, lack of resources for implementation and poor ownership of these strategies.^12^

Therefore, despite the reforms, the revision of the administrative system and the different strategies adopted for retaining the health workforce in the rural districts, most of the rural districts have failed to retain the adequate number of skilled health workers, thus challenging the realisation of the health sector reform goals.^8,15,18^ In Tanzania like in many other parts of the world, the majority of studies on health workforce issues have focused on describing the problem, such as documenting the shortages of health workers and geographical imbalances, and evaluating ‘supply side’ strategies, such financial and non-financial incentives.^6,8,18,22^ Studies of the factors associated with the retention of health workers in rural Tanzania suggest that the ability to settle in a rural community may be more important than job or post characteristics.^23^ However, there is a dearth of literature on how local communities and their actions influence the retention of health workers in rural areas. The aim of this study was to explore the role played by local communities in the retention of health workers in rural Tanzania.

## Methods

###  Study Design

 This exploratory qualitative study was conducted in two rural districts in Tanzania between 2015 and 2016. The exploratory study design was considered appropriate as the role of communities in the retention of health workers was scarcely known and yet thought to be embedded in the social processes of communities.

###  Study Setting and Site Selection

Tanzania operates a decentralised health sector administrative system that is organised in a pyramid of three levels: (*i*) the primary level, comprised of the district health management (Council Health Management Team and District Health Services Board), district hospital, health centres, dispensaries and in some areas, a community health post. At the primary level, there is also an organised community-based health system providing the link between primary health facilities and the local communities through the CHWs^16^; (*ii*) Secondary level which comprises the regional and regional referral hospitals; and (*iii*) Tertiary level which constitutes the zonal referral hospitals, specialised hospitals, and the national hospitals. This study was conducted in two rural districts, one in the Kilimanjaro region, northern Tanzania (herein referred to as A) and the other in the Lindi region, southern Tanzania (herein referred to as B). This East African country is subdivided into seven geopolitical zones (Table 1).

**Table 1:** Geopolitical Zones of Tanzania

**Zone**	**Regions**
Central zone	Dodoma and Singida
Eastern zone	Coast, Dar es Salaam and Morogoro
Lake zone	Kagera, Mara, Mwanza, Shinyanga, Simiyu and Geita
Northern zone	Arusha, Kilimanjaro, Manyara and Tanga
Southern zone	Lindi and Mtwara
Southern highlands	Iringa, Mbeya, Ruvuma and Njombe
Western zone	Katavi, Kigoma, Rukwa and Tabora

The two zones (Figure) were purposefully selected to explore the influence of the different cultural practices, economic activities and the availability of social services. In the southern zone, Lindi was purposefully selected among the two regions forming that zone. This zone is amongst those with the most severe health workforce shortages.^24^ From the northern zone, the Kilimanjaro region was purposefully selected to represent the zones with more favourable health workforce availability in the country.^24^ The two districts (A and B), one from each zone, were randomly selected from a pool of six districts forming each region. The main economic activities of people in district A are small scale farming, livestock keeping, business and tourism, and the majority of the population are Christians. In district B the main economic activities are similarly small scale farming, fishing, business and tourism. The majority of the residents in district B observe the Muslim faith. Two wards were randomly selected from the 25 five wards in district A, and two wards were randomly selected from the 20 wards district B. From the selected wards in the two districts, all villages with health facilities were included in the study (Figure).

**Figure:**
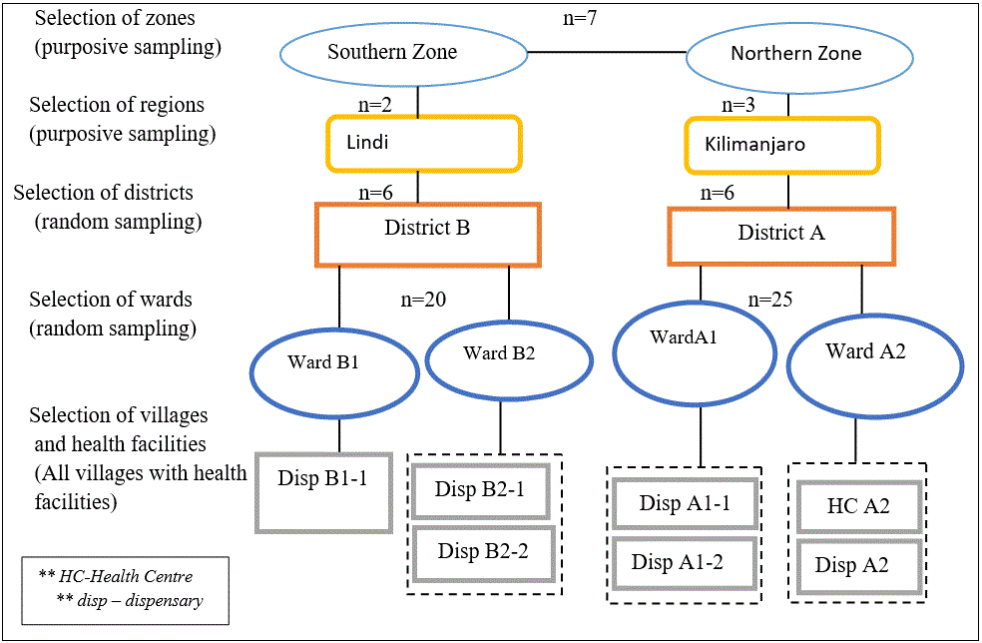
Selection of Study Sites.

###  Selection and Recruitment of Study Participants

 We used purposive sampling to identify the key informants and focus group discussion (FGD) participants by their positions and responsibilities concerning health worker retention. From the two districts, informants were selected from the district to the dispensary level in the selected wards and villages. They included District Medical Officers, District Health Secretaries, District Executive Directors, the District Hospital Medical Officers in charge, Health Centres’ medic in-charge, Ward Executive Officers, dispensaries’ Clinical Officer-in-charge and the Village Executive Officers. The study also involved FGDs with members of the HFGCs of the selected facilities.

###  Data Collection

Using a Kiswahili semi-structured interview guide and a FGD guide, we conducted 19 key informant interviews (KIIs) and three FGDs with 19 members active in the HFGCs from the selected facilities (Table 2). The first author conducted all interviews and moderated the FGDs and a research assistant took notes during the interviews and discussions. All KII and FGDs were audio-recorded using a digital voice recorder. The audio recorder was kept secured by the researcher. The KIIs lasted between 30 and 60 minutes while the FGDs lasted between 60 and 90 minutes. The interviews were carried out in the natural settings of the informants, including their offices or designated rooms at the facilities. The FGDs were carried out at one of the designated rooms at the health facility or at a classroom in a nearby school as identified and arranged by the village leader. The age of participants ranged from 26 years to 58 years with work experience from 2 years to 28 years.

**Table 2:** Participants for KII and FGDs

**Category**	**Male**	**Female**	**Total**
Key informants			
District Executive Director	1	0	1
District Medical Officers	2	0	2
Ward Executive Officers	1	1	2
Village Executive Officers	1	1	2
District Health Secretaries	0	2	2
Health Facility In-charges	4	5	9
Human Resources Officer	0	1	1
FGD participants (members of HFGCs)	11	8	19

Abbreviations: KII, key informant interview; FGD, Focus group discussion; HFGCs, Health Facility Governing Committees.

###  Data Analysis

 All interviews and FGD transcripts were transcribed verbatim and translated into the English language. To avoid loss of the original meaning, back translation was completed for some of the transcripts, especially those that were analysed by non-Kiswahili speakers in the research team, while for native Kiswahili speakers, the analysis was completed using the Kiswahili transcripts and quotes; codes, themes and sub-themes were translated to English. In this analysis process, the District Executive Directors, Ward and Village Executive Officers were grouped into one category named the ‘Local Government Executive Officers;’ the District Medical Officers, District Health Secretary and the District Human Resource Officer were grouped as ‘Health Managers;’ and the HFGC members were referred to as FGD members.

We used a thematic data analysis approach, using both inductive and deductive reasoning.^25^ First, we started by developing an initial codebook for data analysis, based on our study objectives. We then refined the codebook from the themes which emerged during the analysis. The first author developed the initial codebook and shared it with all authors. The codebook was discussed, further developed, and a final codebook was imported into NVivo 11 qualitative data analysis computer software. Second, we tested the agreed codebook by having three authors independently coding the first two interview transcripts. Their coding was almost identical and, hence, the codebook was not modified at the time. At this stage, although the data analysis was guided, it was not confined to the primary codes.

 Third, inductive coding was assigned to text segments which represented a new theme that was not pre-determined. The new codes were assigned as separate codes or an expansion of the codes available in the initial codebook. Fourth, through comparisons, checking similarities and differences, the codes were sorted into categories that were further clustered into sub-themes and then aligned into themes. The whole process of analysis was iterative, with further scrutiny carried out by going back to the interview transcripts to identify, summarise, and retain the patterns and similarities, differences and newly emerged themes. Finally, we presented the themes with supporting and succinct quotes that describe the meaning underpinning each theme.

## Results

From 19 KIIs and 3 FGDs with 19 participants, two overarching themes emerged: accommodation of the health workers into the local communities and the rejection of health workers by the local communities (Table 3).

**Table 3:** Roles Played by the Community in the Retention of Health Workers

**Theme**	**Sub-themes**
Accommodation of the health workers into the local communities	Good community reception of new health workers Local government engagement in resolving challenges facing the health facilities and health workers Local government leaders acting as a link between health workers and the local communities The value placed by the community on the health workers
Rejection of health workers by the local communities	Lack of trust in health workers by the community Cultural rituals practiced by the local community Discrimination of the health workers by the local communities due to cultural differences

###  Accommodation of the Health Workers into the Local Communities 

 Accommodation of health workers refers to the actions that the communities undertook to ensure the absorption and acceptance of health workers hailing from outside their district into their host communities. These actions were viewed as essential in promoting the retention of the health workers in their work posts by making them feel a part of the community. The actions included good community reception of new health workers, local government engagement in resolving challenges facing the health facilities and health workers, local government leaders acting as a link between health workers and the local communities, and the value placed on health workers by the local community members.

####  Good Community Reception for New Health Workers

 Good community reception of the newly employed or transferred-in health workers was reported by some health facility in-charges as contributing to the retention of health workers in their facilities. Health facility in-charges reported that in collaboration with the local government leaders, they organised a welcome ceremony for the newly employed or transferred-in health workers. These ceremonies brought together community members from different villages, the community (village) leaders, and the newly employed or transferred-in staff. Opportunity was taken to introduce the new health workers to the community. Sometimes, this introduction process was taken further to include the introduction of new health workers to members/heads of households. This event made the incoming health workers feel valued by their host community:

*“…we received one clinical officer last year, the village chairperson took him around and introduced him to the community…the community was very happy, he also felt good for such a positive reception…”* [Health facility in charge-4-District B].

 In district A, some facilities had made internal arrangements for an annual ceremony. In this ceremony several activities took place. This included giving a prize to the best performing health worker, and inviting and introducing new health workers to other health workers and local government leaders. During this occasion, health workers who were retiring and those transferred to other places were also bid farewell:

*“…in this party we welcome and introduce newly employed or transferred-in health workers in that year, we introduce them officially to the community, we say farewell to retirees and transferred workers … we give prizes to the best performers of the year … all these make our workers feel valued and united…” *[Health facility in charge-6-District A].

####  Local Government Engagement in Resolving Challenges Facing the Health Facilities and Health Workers 

 In district A, local government leaders reported working closely with the health workers, participating in resolving challenges for both the health facilities and the health workers. The local government leaders felt that their engagement in resolving such challenges made the health workers feel valued. This contributed to satisfaction with their work as well as the working environment. Informants further reported that local government leaders achieved this by organising regular meetings with facility in-charges to discuss the day to day running of the health facilities.

*“…our Ward Development Committees and Village Development Committees meet at least once every month to discuss many issues, including the challenges facing our health facilities and our workers*…” [Local Government Executive Officer-2-District A].

####  Local Government Leaders Acting as a Link Between Health Workers and the Local Communities 

 Informants from both districts stated that the fact that local government leaders come from the community and work closely with the health workers, contributed to accommodation by providing a link between health workers and communities. It was further reported that the engagement of the local government as representatives of the community in the day-to-day matters of the health facilities minimised friction between the health workers and the community, thus providing a supportive environment for work and living to newly employed or transferred-in health workers:

*“…initially, it used to be a challenge, every day it was complaints from the community about our services here … we stood our point, they stood theirs and frictions were always there…. But when we realized the importance of engaging their leaders and thus formulation of the committees, the committee has acted as an important linkage and it clears most of the issues before getting into frictions…” *[FGD member-3-District B].

####  Value Placed by the Community on the Health Workers

 Members from the HFGCs from district A reported putting great value on the health workers’ contribution to their communities. According to them, health workers’ attrition puts the community at risk of not getting health services and communities were advised by their local leaders to ‘handle health workers nicely,’ to make sure that they are retained. The HFGC participants further added that supporting health workers with some of their daily chores showed they valued them and made them feel part of the community. The latter was mentioned as contributing to conducive health workers’ work and living environments:

*“…the health workers are there for us and we value them, for instance, this Saturday we (the community) will be cleaning the surroundings of the Health Centre … they take care of our health we must take care of them so as they stay with us…”* [FGD member-6-District A].

###  Rejection of Health Workers by the Community 

 Health managers, health facility in-charges and members of the community stated that sometimes the community rejected health workers, forcing them to seek transfer to other places. The common forms of rejection were open expressions of lack of trust in health workers by the local community through their leaders, indirect rejection of health workers through practices referred to as ‘superstition’ and discrimination of the health workers by the local communities due to cultural differences.

####  Lack of Trust in Health Workers by the Community 

 Health managers from district B reported that some communities, through their community leaders, expressed lack of trust in health workers. Although the reasons for this lack of trust were not always clear, the managers felt that the community wanted health workers who originated from district B and that those who came from outside the district were pejoratively termed as ‘foreigners.’ The managers added that this situation made it difficult to retain health workers in the district as it created uncertainties and fear for the health workers:

“…*for instance, there were two health workers in one facility, an old woman and a young lady, the political leader came to my office and said that they (the community) didn’t want the young lady…a few days later the young lady came and said the leaders didn’t want her and they are embarrassing her. So, she decided to leave…” *[Health manager-1-District B].

 Members of the HFGCs in district B added that sometimes the lack of trust in health workers by the community was attributed to poor engagement with community leaders by the health facility managers on matters involving the running of the facilities. In the FGD with a health committee in one ward, the majority of the discussants reported that they were not aware of, or involved in, issues concerning the health facilities in their villages:

“…*there is no involvement in the health services board at the ward level and below…this makes us as a community see everything as a new thing, sometimes you decide to go to the health facility and get a surprise from everyone there…it is called a government facility but as representatives of the community we are not involved in anything…”* [FGD member-2-District B].

####  Practice of Cultural Rituals by the Local Community

In district B, health managers reported that health workers perceived themselves to be at the receiving end of practices they referred to as *‘cultural rituals’* from the local community. These practices were described as mostly affecting the very remote areas and as grounded in local cultural beliefs. Health workers who came from outside district B and who had not encountered such practices before, found them hard to accommodate. They added that some of these practices were seen by health workers as instigating fear of the unknown or witchcraft. The latter was reported to represent an indirect but powerful form of community rejection. The managers indicated that in such instances, health workers were quick to seek transfers to places out of district B or in semi-urban areas within this district, adding that if the opportunity for transfer was denied, health workers would opt to resign instead.

*“…this community practices a lot of cultural rituals … when it happens, a worker will come and tell you, I have failed, I can’t stay here with these witches, give me a transfer or I resign … now as you know the situation you give her/him the recommendation letter for transfer…” *[Health manager-2-District B].

####  Discrimination of the Health Workers by the Local Communities Due to Cultural Differences

 Wider cultural differences between health workers and communities were cited among the major challenges of health worker retention in district B. Cultural differences were based on tribal origin and further complicated by the differences in religious beliefs. New health workers that did not originate from district B stated that they had difficulty securing houses for rental. Landlords were more comfortable renting their houses to people who shared the same culture as them, and in the event health workers were able secure a house, they experienced living with indigenous tenants as challenging.

 Similar problems were reported in district A, where health workers indicated being denied access to the purchase of land for building and cultivation by their host communities. The indigenous members of the communities were not ready to sell their pieces of land to health workers, especially those not originating from the area. For this reason, some of the health workers opted to seek transfer to other places:

*“…. Here people have placed the value of their land very high, it is at least easier to sell to someone who originates from here but it is extremely difficult if you are not from here … even if it were you, how can you live like a tenant throughout your life?”* [Health Manager -2-District A].

 In places where religious beliefs were strong, health workers not belonging to the religion of the majority were excluded from community social activities. Alongside the cultural practices described earlier, these factors were regarded as major reasons for seeking transfer or resigning.

*“…here most of the people are so religious…. If you do not belong to their religion, they leave you aside even in community social activities, how do you stay in a community while feeling excluded?” *[Health facility in charge-3-District B].

## Discussion

 We aimed to explore the role played by local communities in the retention of health workers in rural areas. The findings from this study shed light on how local communities can accommodate or reject health workers through different means. The largely contrasting experiences of accommodation and rejection in the high (A) and low (B) retention districts, respectively, suggest that community factors have an important bearing on rural health worker retention. While accommodation promotes the retention of health workers, rejection fuels their attrition. The latter is attributed to the role of the local community and health workers’ relationship and actions of the local community.

###  Role of the Relationship Between the Local Community and Health Workers in the Retention of Health Workers

The findings of this study resemble findings of other studies that have highlighted the importance of the relationship between local communities and health workers in the retention of health workers in Tanzania and other places. Shemdoe et al, in a 2016 study explaining the retention of health workers in Tanzania, documented that better retention of health workers in rural areas was related more to settling into the community than into particular job postings.^23^ From the findings of our study, we explain settling into the community as the interaction between the community and the health workers, involving principally the acceptance of health workers into the cultural norms of the local community. Acceptability and the integration that follows enhance ties between health workers and the local communities. This is similar to the findings of Kiwanuka et al in Uganda.^26^

From the findings of our study, how the community receives and treats the health workers has major bearing on the relationship between them. Community attitudes crucially reflect the value placed by the community on health workers and is vital in their retention. When health workers feel a sense of being valued it creates a sense of belonging and they are more likely to stay and work in that particular community. Similar observations have been made in Bangladesh and Australia. In a study conducted in Bangladesh, it was reported that the retention of CHWs was motivated, among other factors, by the value placed on them by the community.^27^ In rural Australia, overseas trained doctors who were returning home were motivated to stay in rural communities due to the welcoming culture of the local communities.^28^ Similar to our findings, the latter study stressed further the importance of good reception of the health workers by the local community.

Brunton et al^29^ have highlighted the crucial role of community engagement in the effectiveness of public health interventions. Community engagement is viewed to increase the acceptability of public health interventions and enable mutual learning. In our study, when the community was engaged and integrated into the running of the local health services, a sense of trust was created that made it easier to accept and accommodate new incoming health workers to their community. In a Zambian study on the engagement of the community in informed consent, Zulu et al^30^ describe community participation as complex and influenced by socio-cultural values, but that undermining this participation may result in mistrust and affect implementation of interventions.

###  Actions of the Local Community and Retention of Health Workers

Our study has revealed that where the local government leaders and the community participate in addressing the challenges facing the health workers and health facilities, these actions promote a sense of togetherness between the local community and the health workers. In a study conducted in rural Nigeria, it was revealed that the actions of communities may constitute important retention factors that can overcome the effects of push factors such as erratic financial and non-financial incentives.^31^ This study concluded that the communities play an important role in retaining health workers through the provision of social, financial and accommodation support. In another Nigerian study by Ebuehi and Campbell, a better living environment and family support systems were reported to motivate the health workers to stay in rural areas.^32^

 From the findings of this study, we argue that it is the relationship between the health workers and the local communities that can enhance the retention of health workers. We further argue that the local community leaders have a large role in linking and integrating the health workers with the local communities and thus can foster good relationships, contributing to the retention of health workers. In the same manner, local community leaders can foster a bad relationship between the health workers and the local communities and thus contribute to the poor retention of health workers. In Tanzania and perhaps in many other resource-constrained countries, building on better community relationships with health workers can perhaps be a feasible and sustainable option in promoting retention of health workers in rural areas compared to financial incentives.

We further interpret the findings of our study in line with Vroom’s expectancy theory of motivation.^33^ According to Vroom, when the worker has the correct support (expectancy) to do the job, s/he is likely to do it better. Vroom adds that when the job done by this worker is valued (instrumentality) there is a chance of increasing performance in the expectation of more rewards (valency). From our study findings, when the community places value on the health workers by supporting them as revealed, it adds to their feelings of being valued and thus motivated to work for this community. When a worker is motivated to work for a particular community, this worker will likely stay and stick in that community. Bonenberger et al^34^ explain the relationship between health workers’ motivation, satisfaction and turnover in Ghana, pointing out that motivated and satisfied health workers are more likely to be retained at the district level than their counterparts.

Conversely, our study has also revealed that the community may at some point reject the health workers. The rejection may be direct or indirect by providing an unfavourable living environment to the health workers in the community. The creation of a hostile environment through the local community view of health workers as foreigners was also documented in a study by Sirili et al on the retention of medical doctors in Tanzania.^35^ Similar to the findings of this study, some communities made it hard for health workers to acquire a piece of land or even a house for rental in order to settle in the area. The role played by access to accommodation is also recognised in several other studies on enhancing the retention of health workers.^6,7,35,36^ When health workers are uncertain of their accommodation and safety, the role that can be played by monetary incentives is highly limited. Poor retention of health workers caused by hostility to non-indigenous health workers deployed to work in rural and remote regions without prior consultation with local authorities has also been described by Awofeso in rural Nigeria.^37^ Community engagement in a truly grounded manner can assist in addressing challenges related to community rejection of health workers. This can be achieved by increasing trust between health workers and communities through building authentic partnerships which include mutual respect as well as active and inclusive participation.^38^

###  Study Limitations

 The fact that a health worker (medical doctor) led the interviews might have induced social desirable responses from the participants. However, the triangulation of informants from all levels of the health service and the community, the settings selected for interviews and having research assistants with social science backgrounds offset this limitation. The majority of participants in the study represented local elites, whether from the health department or local government, and the narrative may be biased to reflect their perspectives. The inclusion of community members in the FGDs partially offsets this limitation.

## Conclusion

 The findings of this study underscore the role of the relationship between the local community and health workers, and the community actions on the retention of health workers in rural areas. Investing in building a good relationship between the local community and health workers, and fostering actions which promote the reception and integration of health workers in the community could be a worthwhile investment. Positive relationship building requires collaborative efforts from health workers, community members and local leaders. Promoting and creating social spaces for engagement can be used as a strategy by local government authorities to nurture positive relationships. Rural communities should be engaged in designing locally acceptable but yet feasible interventions in promoting the retention of health workers in their communities. The various roles communities could play in rural health worker retention is worthy of further research.

## Ethical issues

 Ethical clearance for this study was obtained from the Senate Research and Ethics Committee of the Muhimbili University of Health and Allied Sciences in Tanzania (Ref. No. 2015-01-15/AEC/Vol.IX/43). Permission for data collection was obtained from the ministry responsible for health, the ministry responsible for local government, regional and district authorities, and heads of health facilities. Written informed consent was obtained from each study participant before the KII or FGD.

## Competing interests

 Authors declare that they have no competing interests.

## Authors’ contributions

 NS, lead the drafting of the manuscript, DS, JMZ, and GF provided supported NS in the drafting of the manuscript and provided technical guidance. MT provided overall guidance to the drafting of the manuscript and its revision. All authors reviewed and approved the manuscript.

## Authors’ affiliations

^1^Department of Development Studies, School of Public Health and Social Sciences, Muhimbili University of Health and Allied Sciences, Dar es Salaam, Tanzania. ^2^Department of Community, School of Public Health and Social Sciences, Muhimbili University of Health and Allied Sciences, Dar es Salaam, Tanzania. ^3^School of Public Health, University of Zambia (UNZA), Lusaka, Zambia. ^4^School of Pharmacy, University of Waterloo, Waterloo, ON, Canada. ^5^Department of Epidemiology and Global Health, Umeå University, Umeå, Sweden. ^6^Department of Health Policy, Planning and Management, Makerere University School of Public Health, Kampala, Uganda.
